# Meeting of the Central Michagan Dental Association

**Published:** 1872-08

**Authors:** 


### Meeting of the Central Michagan Denal Association.

The Central Michigan Dental Association met July 10th, 1872, in the parlors of the Lawrence House, in Adrian, and was called to order by the President, Dr. C. C. Jenkins, of Ann Arbor.

The Secretary, Dr. W. H. Dorrance, of Jackson, called the roll of members, and those present answered to their names.

The minutes of the last meeting were read and approved.

Drs. Taft, of Cincinnati; Clippinger and Harroun, of Toledo, were elected honorary members.

Dr. M. H. Knapp, from the committee appointed for the purpose, announced that the services of Dr. Taft, of Cincinnati, had been secured during these sessions. The report was accepted and adopted.

Under the head of miscellaneous business the convention held a general discussion on various professional topics, first canvassing the merits of Guillois' cement.

Dr. Jackson, had used Guillois' cement, but had used more of the oxychloride of zinc, of Roberts' make. He thought the former preferable to the latter for some reasons, and was sometimes easier to use.

Dr. Dorrance, of Jackson, had not been fortunate in the use of Gullois' cement. He thought perhaps he had been imposed upon by an inferior article. He complained of its appearance.

Dr. Jackson, did not believe the cement would be found suitable for permanent filling, and he had found that no material of that nature would in his opinion be suitable.

Dr. Dorrance, complained that he found the cement did not harden in good shape. It had an oily, soaked appearance.

Dr. Jenkins, of Ann Arbor, had used several samples of the cement, and had found no difficulty with any of them. He preferred it to oxychloride of zinc.

Dr. Knapp, of Fort Wayne, preferred the cement so far as he had used it. He had used it as os-artificial, and though he had used it to a limited degree, he liked it much.

Dr. Taft, of Cincinnati, had used Gullois' cement for two years, and in his practice it had almost entirely superseded the use of os-artificial. He was of the opinion that an inferior article, made in this country, was being introduced to the profession. He had found no difficulty in using the genuine cement. He had used no other. He thought the cement was preferrable to anything else used in treatment, but not for permanent filling. He had used it under gold in filling up a large cavity. He regaided such a filling as equal to one of gold entirely. But neither the cement or oxycloride of zinc should be brought in contact with an exposed pulp. He used the Gullois cement in preferrence to all other material. It was a mistake to lay down a rule and never discriminate. That savored of empiricism. Cases should be treated with careful judgment.

In answer to a question by Dr. Clippinger, Dr. Taft stated that he used the cement of a consistency of soft butter.

Dr. Clippinger, stated some experience of a patient of his, who had an inflamed tooth which had been filled with gold. He removed the filling, and found the pulp protruding. He cut off the protruding pulp, touched the wound with carbolic acid, and used Hill's stopping in temporary filling. . The results had been most satisfactory. The pulp was alive.

Dr. Jackson, had used cotton in the apex of the root, the cotton touched with carbolic acid, and then partly filled the cavity with gold, following with oxychloride of zinc. In this way he had obtained good results.

Dr. Dorrance, had met with good results without using cotton or gold in the apex. He used the oxychloride of zinc from the apex of the root. He thought the application of carbolic acid injurious.

Dr. Taft said he had taken out a pledget of cotton from a tooth which had remained in the cavity for ten years, and at the end of that time the cotton retained the odor of creosote or carbolic acid, quite plainly. He explained the action of the acid in the cavity. He would never use a pledget of cotton in a case where the pulp was living.

Dr. Owen, of Adrian, had had similar experience with Dr. Taft. He had last week extracted a tooth filled five years ago, and cotton and creosote applied to the apex. He found the odor of the creosote as strong as when first applied.

Dr. Harroun, explained his plan of using any of the artificial bone mixtures. He favored carbolic acid, but thought the greatest judgment should be used. He scarcely ever used any of the os-artificial preparations except as a temporary bridge to something better.

Some discussions ensued on the matter of sensitive dentine. Dr. Taft attributed it to the action of local irritating agents, together with systemic predispositions, and its cure is to be found in the removal of the irritants and the improvement of systemic conditions, in most instances, merely local applications would not remove the sensitiveness. Systemic treatment is necessary. In cases where the sensitiveness arises from local causes, simple local treament will remove the trouble.

The society then adjourned to 7½ o'clock P. M.

To Be Continued.

